# Retroperitoneal malignant peripheral nerve sheath tumor treated with laparotomy approach: A case report

**DOI:** 10.1016/j.ijscr.2025.110998

**Published:** 2025-02-01

**Authors:** Intan Andaru, Wahjoe Djatisoesanto, Karinda Triharyu Caesari Putri

**Affiliations:** a Department of Urology, Faculty of Medicine, Airlangga University, Dr. Soetomo General Academic Hospital, Surabaya, Indonesia; b Department of Urology, Prof. Dr. Margono Soekarjo Hospital, Purwokerto, Indonesia

**Keywords:** Retroperitoneal tumor, Malignant, MPNST, Laparotomy

## Abstract

**Introduction:**

Malignant peripheral nerve sheath tumors (MPNST) are sporadic neoplasms that present significant diagnostic challenges, particularly in retroperitoneal locations. While these aggressive tumors most commonly occur in the head, neck, and upper extremities, retroperitoneal cases represent a mere 1 % of all instances. This case study examines a specific instance of retroperitoneal MPNST diagnosed and treated through laparotomy, with the primary objective of enhancing medical professionals' understanding of this uncommon tumor's diagnostic complexities, treatment approaches, and potential prognostic implications. By highlighting such a rare clinical scenario, the research seeks to raise awareness among clinicians about the nuanced considerations required when encountering these challenging and infrequent malignancies in unusual anatomical regions.

**Case presentation:**

During a medical investigation of abdominal pain in a 44-year-old female patient, advanced imaging revealed a complex mass located in the left adrenal gland. Computed tomography scans demonstrated significant anatomical involvement, with the tumor compressing adjacent structures, including the pancreas and spleen superiorly, the left kidney and renal vasculature inferiorly, and positioned adjacent to the abdominal aorta. Surgical intervention was undertaken with the objective of complete tumor removal, successfully achieving unambiguous surgical margins. Subsequent immunohistochemical analysis confirmed the diagnosis of an MPNST, providing critical insights into the nature of the patient's complex medical condition.

**Discussion:**

MPNST represents a complex and challenging neoplasm characterized by its highly invasive and rapidly progressing nature, originating from neural tissue. The diagnostic process for MPNST is intricate, primarily due to the absence of definitive histological criteria and a distinctive immune profile. Critical diagnostic challenges emerge from the significant morphological similarities between MPNST and other tumors, such as fibrosarcomas and leiomyosarcomas. In this specific case, pathological anatomy initially suggested a liposarcoma lesion; however, immunohistochemistry testing revealed a negative Desmin result, effectively eliminating the liposarcoma diagnosis and underscoring the nuanced complexity of accurate tumor classification.

**Conclusion:**

This case report highlights the diagnostic difficulty in identifying divergent differentiation in sarcomas, using MPNST and liposarcoma as examples.

## Highlights

• MPNST are rare and aggressive tumors. Moreover, retroperitoneal instances are challenging to diagnose.
• Distinguishing MPNST and other differential diagnosis, such as fibrosarcomas and leiomyosarcomas, is crucial.
• This study highlights the diagnostic difficulty of MPNST and attempts to raise awareness regarding its diagnosis, treatment, and prognosis.

## 1 Introduction

Malignant peripheral nerve sheath tumors (MPNST) represent a rare and complex soft tissue sarcoma, constituting approximately 3–10 % of all sarcomas. This distinctive neoplasm predominantly emerges in patients aged 20 to 50, with approximately half of the cases occurring in individuals diagnosed with neurofibromatosis type 1 (NF1). Characterized by its origin in peripheral nerves, the tumor demonstrates variable cellular differentiation across the nerve sheath's constituent elements. While most frequently manifesting in extremities, MPNSTs present significant diagnostic and clinical challenges due to their uncommon nature and complex pathological characteristics. The tumor's rarity and potential for aggressive progression underscores the importance of specialized medical expertise in its identification and management [1,2].

The few non-NFL-associated instances usually occur spontaneously without any detectable benign precursor lesion. Occasionally, MPNSTs have developed in schwannomas or ganglioneuromas. The diagnostic histologic characteristics include tightly packed and loosely packed bundles of fibers, spindle-shaped cells that taper unevenly, cells with nuclei that seem buckled or wavy, arrangement of nuclei in a palisading pattern, structures that are arranged in a whorled fashion and an increased number of cells around blood vessels. The tumor cells demonstrate characteristics of Schwannian differentiation through immunohistochemistry, specifically staining positive for S100, collagen IV, and laminin. This is also confirmed by electron microscopy, which reveals the presence of thin cell processes and the deposition of basement membrane material. Unlike benign Schwann cell neoplasms, which consistently exhibit positive S100 staining, MPNSTs display variable staining for S100. Approximately 15 % of MPNSTs have areas of heterologous or divergent differentiation [3]. This case report will present retroperitoneal MPNST with adrenal involvement, adhering to the SCARE criteria [4].

## 2 Case presentation

**Fig. 1:**
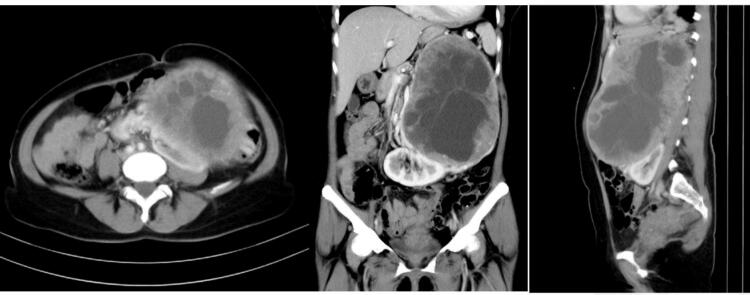
CT scan of the abdomen shows a mass at the upper region of the adrenal.

A 44-year-old female patient presented to the urology clinic with a progressively enlarging abdominal mass, which had been developing over the past six months. Her clinical symptoms included significant gastrointestinal disturbances: persistent nausea, early satiety, diminished appetite, and notable unintentional weight loss of 5 kg during the preceding three-month period. Despite the apparent systemic changes, the patient reported no accompanying cardiovascular, neurological, or endocrine symptoms such as palpitations, headaches, or excessive sweating. Her medical history was unremarkable, with no documented comorbidities or prior surgical interventions, further complicating the preliminary diagnostic assessment. (See Fig. 1.)

On examination, vital signs were within normal limits, and a hard, painless lump measuring approximately 15 cm × 10 cm was palpated in the left upper abdominal region. Routine laboratory tests were regular. Ultrasonography revealed an inhomogeneous mass in the upper abdominal region that exceeded the probe. Computed tomography (CT) scan showed a 13.8 12.7 × 19.1 cm solid object attached and difficult to separate with sinistra adrenal gland, urging pancreas and lien to the superior, ren sinistra and a/v.renalis sinistra to the inferior and abdominal aorta to the dextra which was suspected to be an adrenal mass or metastasis as seen in Fig. 1. The patient underwent surgical resection of the left adrenal with a lumpectomy approach. Adrenal tumor removal was performed quite smoothly with 100 cc bleeding. Postoperative care was performed for five days. The postoperative wound closed well after two weeks. There were no significant surgical complications.

An anatomical pathology examination was performed to confirm the diagnosis. On macroscopic examination, the tissue size was 22 × 16 × 12 cm; the division contained blackish coat fluid with cavities. The preparation from the retroperitoneal sinistra showed a hypercellular tumor composed of solid clusters of non-cohesive epithelioid cells among which vascular proliferation appeared, tumor cells partially grew in a perivascular pattern, tumor cells with oval, round nuclei and hyperchromatic spindles, moderately mitotic, granular cytoplasm. Hemorrhage and necrosis were observed. Some multinucleated data cells were seen with the impression of Perivascular epithelioid cell tumor (PEComa). The pathologist suggested a follow-up CEC examination. Immunohistochemical (IHK) leads to Malignant Melanoma dd MPNST and liposarcoma: Advanced IHK HMB 45, SOX 10, desmin, and myogenin show negative results. The patient's post-surgical treatment strategy comprises radiotherapy and chemotherapy, with a systematic imaging surveillance protocol designed to monitor and identify potential metastatic lesions for proactive medical intervention.

## 3 Discussion

MPSNT represents an exceptionally challenging neoplasm characterized by its aggressive neural tissue origin and rapid progression. The diagnostic complexity of this tumor stems from the lack of definitive histological markers and a distinctive immunological profile, which significantly impedes accurate clinical identification and classification. It is crucial to distinguish between some tumors, such as fibrosarcomas and leiomyosarcomas, and MPNST due to their similar physical characteristics. Fibrosarcomas exhibit a straightforward organization, characterized by a high concentration of collagen and a lack of S-100 protein when examined by immunohistochemistry. Leiomyosarcoma cells display vacuoles surrounding the nuclei. These abnormalities form in tissues and organs with a high concentration of smooth muscle. Diagnosing malignant peripheral nerve sheath tumors (MPNST) requires sophisticated immunohistochemical analysis to distinguish them from other neoplasms, mainly when epithelioid cells are prevalent. While demonstrating positive staining for desmin and smooth muscle actin, these tumors necessitate careful differentiation from malignant melanoma and epithelioid sarcoma through nuanced morphological and molecular markers. Malignant melanoma can be distinguished by its more superficial lesion location, irregular cell shape, a larger nucleus with intense staining, and positive HMB45 and Melan-A markers. In contrast, abundant collagen fibers characterize epithelioid sarcomas and notably lack neuron-specific enolase staining, providing critical diagnostic insights for accurate tumor classification and subsequent clinical management [5,6].

.The tumors were primarily made up of spindle cells that were reasonably uniform in shape and grouped in intersecting long bundles. The cells in the lesion displayed ovoid nuclei and had a high degree of chromaticity. The nucleoli were not easily noticeable, and the cytoplasm was minimal. MPNST is commonly classified as a high-grade sarcoma. The current investigation found a relatively high mitotic score consistent with this classification. The Desmin testing yielded negative results in this patient, ruling out the possibility of a liposarcoma diagnosis [7,8].

According to medical literature, chemotherapy offers minimal improvement in patient survival rates and is primarily utilized in advanced cancer stages, specifically for metastatic conditions [9]. The critical determinants of overall patient survival encompass three primary independent factors: the tumor's grade and size and the technical proficiency of surgical interventions [10]. MPNST demonstrates substantial clinical challenges, with documented local recurrence rates ranging from 40 to 65 % and metastasis potential between 30 and 60 %. The primary risk factors for both local recurrence and metastatic progression are primarily characterized by tumor dimensions and staging characteristics, with tumors larger than 10 cm significantly elevating the probability of adverse outcomes. Surgical intervention plays a critical role, as positive surgical margins substantially increase the likelihood of local recurrence. At the same time, tumors classified under stage III by the American Joint Committee on Cancer carry an elevated risk of metastatic spread [11].

## 4 Conclusion

This case report highlights the diagnostic difficulty in identifying divergent differentiation in sarcomas, using MPNST and liposarcoma as examples. Although all cells in a tumor originate from a single cell through clonal proliferation, there can be significant variances in the phenotype of various cells within the same tumor. The differentiation steps are typically viewed as irreversible decisions in a branching process leading to a particular cell phenotype. Explaining distinct neuronal or epithelial features in sarcoma using a branching differentiation model is challenging. Cell differentiation may not always be a strictly unidirectional process.

## Consent

The patient provided formal written consent to publish this case report and associated medical images. Upon request, a copy of the written authorization is available for the Editor-in-Chief's review.

## Provenance and peer review

This manuscript was not commissioned and has undergone external peer review.

## Ethical approval

Ethical clearance for this case report was obtained from the Hospital Research Ethics Committee, where the patient received treatment.

## Funding

No financial funding was received for this article's research, authorship, or publication.

## Author contribution

Intan Andaru: Investigation, Methodology, Resources, Writing - original draft.

Wahjoe Djatisoesanto: Conceptualization, Supervision, Project administration, Writing - review & editing.

Karinda Triharyu Caesari Putri: Conceptualization, Methodology, Writing - original draft, Writing - review & editing.

## Guarantor

Wahjoe Djatisoesanto.

## Research registration number

NA.

## Declaration of competing interest

The authors affirm no conflicts of interest relevant to this publication.
